# Computational Study of Quenching Effects on Growth Processes and Size Distributions of Silicon Nanoparticles at a Thermal Plasma Tail

**DOI:** 10.3390/nano11061370

**Published:** 2021-05-21

**Authors:** Masaya Shigeta, Yusuke Hirayama, Emanuele Ghedini

**Affiliations:** 1 Graduate School of Engineering, Tohoku University, Sendai 980-8579, Japan; 2 National Institute of Advanced Industrial Science and Technology, Nagoya 463-8560, Japan; hirayama.yusuke@aist.go.jp; 3 Alma Mater Studiorum, University of Bologna, 40126 Bologna, Italy; emanuele.ghedini@unibo.it

**Keywords:** quenching, nanoparticles, growth, plasma, multiscale modeling and simulation

## Abstract

In this paper, quenching effects on silicon nanoparticle growth processes and size distributions at a typical range of cooling rates in a thermal plasma tail are investigated computationally. We used a nodal-type model that expresses a size distribution evolving temporally with simultaneous homogeneous nucleation, heterogeneous condensation, interparticle coagulation, and melting point depression. The numerically obtained size distributions exhibit similar size ranges and tendencies to those of experiment results obtained with and without quenching. In a highly supersaturated state, 40–50% of the vapor atoms are converted rapidly to nanoparticles. After most vapor atoms are consumed, the nanoparticles grow by coagulation, which occurs much more slowly than condensation. At higher cooling rates, one obtains greater total number density, smaller size, and smaller standard deviation. Quenching in thermal plasma fabrication is effectual, but it presents limitations for controlling nanoparticle characteristics.

## 1. Introduction

Nanoparticles offer unique electronic, optical, magnetic, and catalytic properties that differ from those of larger micrometer-size particles and those of bulk materials [1]. Nanoparticles are therefore highly attractive for use in applications expected to lead to breakthroughs in biomedical, environmental, and industrial fields. Even pure silicon nanoparticles are anticipated as promising materials for use in numerous applications such as lithography [2], electronic devices [3], electron transistors [4,5], floating gate memory devices [6,7,8,9], luminescent thermometers [10], and lithium ion battery electrodes [11,12,13]. Several Japanese groups have recently used thermal plasmas to achieve high-throughput production of silicon nanoparticles for lithium ion battery electrodes [14,15,16,17,18].

Thermal plasma, a kind of high-enthalpy plasma, is generated by high-power discharge at around atmospheric pressure. Thermal plasma is a unique fluid with high temperatures not only of electrons but also of heavy species, with high chemical reactivity, intensive light emission, and varying transport properties [19]. It is also an electrically conductive fluid. Therefore, it can be controlled by an electromagnetic field [20,21]. The high temperature of its heavy species, around 10,000 K, can vaporize even materials that have a high melting point or boiling point. In addition, the steep temperature-decrease gradient at its tail plays an important role in rapid conversion from material vapor to nanoparticles. By virtue of these two features, thermal plasma achieves one-step fabrication of nanoparticles with high yield [22]. Indeed, based on the use of inductively coupled thermal plasma (ICTP), Tanaka et al. [23] and Kodama et al. [24] synthesized titanium-dioxide nanoparticles at 6.7–12.3 g min^−1^ using a pulse modulation technique. Ohta et al. [15] produced silicon nanoparticles at 17 g min^−1^ using direct-current plasma jet assistance.

The number and size of nanoparticles are affected by temperature and flow fields in and around thermal plasma through growth processes [25]. Strong cross-correlations between the temperature at an upstream plasma fringe and nanoparticle concentration in a downstream region have been found for a transferred arc plasma system [26,27] and for a non-transferred arc plasma jet in two-dimensional [28] and three-dimensional fields [29] in recent numerical studies using a method suitable for simulating dynamic behaviors of thermal plasma–nonionized gas coexisting flows [30]. Actually, to control temperature and flow fields and to control nanoparticle growth processes in ICTP systems, several experimental studies adopted quenching strategies using a water-cooled coil [31], a water-cooled ball [15,32], direct plasma control by pulse modulation [33], counterflow injection [18,34], and radial gas injection [35,36]. Effects of those quenching methods were also investigated using two-dimensional simulations for practical conditions [37,38,39,40,41,42,43,44,45,46,47]. From a more fundamental perspective, specific effects of quenching on nanoparticle size distributions were investigated numerically using time-dependent models [48,49,50,51]. Although those works explicitly presented results obtained for spatial distributions or size distributions of nanoparticles, none clearly presented an implicit mechanism that dominates nanoparticle growth processes.

This study investigated quenching effects on the growth processes and size distributions of nanoparticles at a typical range of cooling rates at a thermal plasma tail. The investigative method is numerical, using a nodal-type model that expresses a size distribution evolving temporally with simultaneous homogeneous nucleation, heterogeneous condensation, interparticle coagulation, and melting point depression. To validate the present numerical calculations, silicon nanoparticle fabrication is demonstrated experimentally with and without quenching. Results of the study reveal the implicit mechanisms of processes related to conversion from vapor atoms to nanoparticles. Moreover, results clearly illustrate cooling rate effects on the total number, size, and dispersion of the produced nanoparticles.

## 2. Experiment Setup

**Figure 1:**
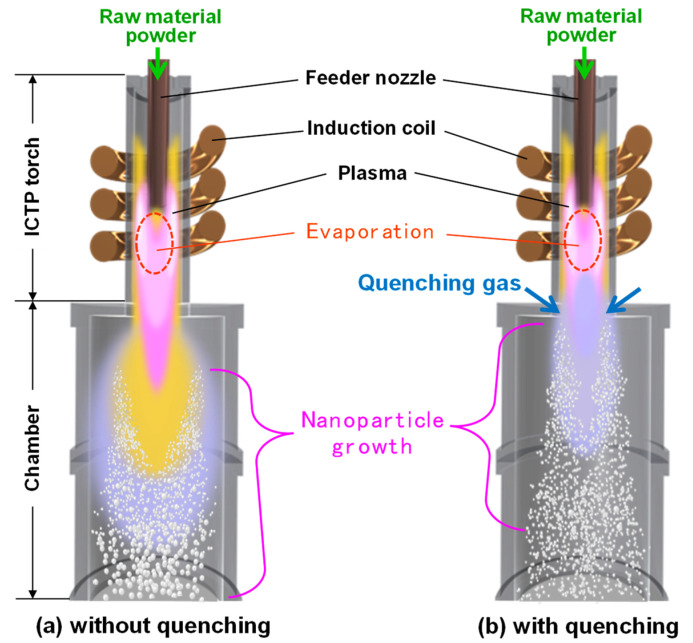
Schematic illustrations of nanoparticle fabrication in ICTP systems (**a**) without and (**b**) with quenching.

Figure 1 portrays schematic illustrations of the nanoparticle fabrication systems (TP-40020NPS; JEOL Ltd., Tokyo, Japan) consisting of an ICTP torch and a chamber without or with a quenching portion. After argon gas (G1 grade, less than 0.1 ppm oxygen) was filled in the main chamber at 100 kPa, argon gas was injected continuously at 35 L min^−1^ from the torch top to sustain the plasma. Using a powder feeding system (TP-99010FDR; JEOL Ltd., Tokyo, Japan), coarse silicon powder (approx. 7 μm particle size, 99.99% purity; Kojundo Chemical Lab. Co., Ltd., Saitama, Japan) was then introduced through the feeder nozzle into the plasma torch at a 0.048 g min^−1^ feed rate, with carrier argon gas added at a flow rate of 3 L min^−1^. When the flow was quenched, additional argon gas was injected from the lower part of the torch toward the central axis at 80 L min^−1^. Details of the system geometries and discharge parameters were identical to those described for an earlier study [52,53].

Observation was achieved by using scanning electron microscopy (SEM: JSM-7800F; JEOL Co., Ltd., Tokyo, Japan). Size distributions for approximately 1200 nanoparticles were measured from SEM micrographs.

## 3. Numerical Model Description

### 3.1. Model Outline and Assumptions

Following evaporation in the plasma, the material vapor is transported through the plasma to the chamber. Vapor reaching the plasma tail experiences a rapid temperature decrease. There, the vapor becomes supersaturated. Numerous nuclei are generated (homogeneous nucleation). Once nucleation occurs, the vapor condenses on the nuclei (heterogeneous condensation). Through these processes, nanoparticles grow. Simultaneously, the nanoparticles mutually collide and merge to form larger nanoparticles (interparticle coagulation) if they are still liquid and exist at temperatures higher than their melting points.

Those nanoparticle diameters vary widely from sub-nanometer scale to a few hundred nanometers, which correspond to monomer numbers in a single particle from 10^1^ to 10^8^. Furthermore, the spatial number density of the nanoparticles reaches 10^18^ m^−3^. Therefore, molecular dynamics calculations for every nanoparticle’s growth are impossible in a practical sense. Aerosol dynamics modelling makes it possible to calculate the collective and simultaneous growth processes of the numerous nanoparticles. Based on the aerosol representation, nanoparticle growth processes at thermal plasma tails have been predicted numerically using several computational models with different features summarized in Table 1. The models are classified in four types. Type A is a sophisticated model with a simple mathematical description treating nanoparticles’ growth by simultaneous homogeneous nucleation and heterogeneous condensation; however, it does not take interparticle coagulation into account [51,54,55,56]. It can express a size distribution with any shape so that it needs high costs of computational resources and time. Type B describes collective nanoparticles’ growth by simultaneous nucleation, condensation, and coagulation by a simple set of two aerosol equations and one vapor equation so that the computational costs are low [26,27,28,29,57,58]. However, it does not express a dispersed size distribution because it calculates physical quantities by averaged size approximation. Type C has been an often-used model which deals with collective nanoparticles’ growth by simultaneous nucleation, condensation and coagulation as well [39,40,42,43,44,45,46,47,59,60,61,62]. Although its computational costs are low, its mathematical formulation is more complex; nevertheless, it still requires an assumption of a lognormal size distribution. Type D can express a size distribution with any shape during collective nanoparticles’ growth through simultaneous processes of nucleation, condensation, and coagulation [37,48,49,53,63,64,65,66,67,68,69,70,71,72,73]. Its mathematical description is more complex; furthermore, its computational costs are higher because the equations as many as the nodes discretizing the size distribution.

This study adopts Type D with several assumptions: (i) spherical nanoparticles; (ii) negligible nanoparticle inertia; (iii) identical transport velocity and temperature of nanoparticles to those of the bulk gas flow; (iv) negligible heat generation caused by condensation; (v) negligible electric charge of nanoparticles; (vi) material vapor regarded as an ideal gas; and (vii) atmospheric pressure in the reaction chamber. Actually, Model type D discretizes the size distribution in the space of particle sizes expressed by particle volume. The discretized volumes are assigned as

(1) $$v_{k+1}=f_{q}v_{k}\;\;\;\;\left(k=1,2,...,k_{max}\right)$$

where v represents the particle volume, k stands for the discrete point number for the particle size, and $f_{q}$ is a coefficient to determine the interval. $f_{q}$ = 1.16 and $k_{max}$ = 161 are set for this study. The minimum volume of a “particle” is chosen as $v_{2}=10v_{1}$, where $v_{1}$ denotes the volume of a monomer, which is equivalent to a vapor atom. In this size space, the net production rate of particles with volume $v_{k}$ is written as

(2) $$\frac{\Delta N_{1}}{\Delta t}={\dot{N}}_{k}^{\left(nucl\right)}+{\dot{N}}_{k}^{\left(cond\right)}+{\dot{N}}_{k}^{\left(coag\right)}\;\;\;\;\left(k=2,...,k_{max}\right)$$

where $N_{k}$ represents the number density of particles at node *k*, $\dot{N}$ signifies the increment per unit time, and superscripts (*nucl*), (*cond*), and (*coag*) stand for the contributions of homogeneous nucleation, heterogeneous condensation, and interparticle coagulation, respectively.

### 3.2. Homogeneous Nucleation

**Figure 2:**
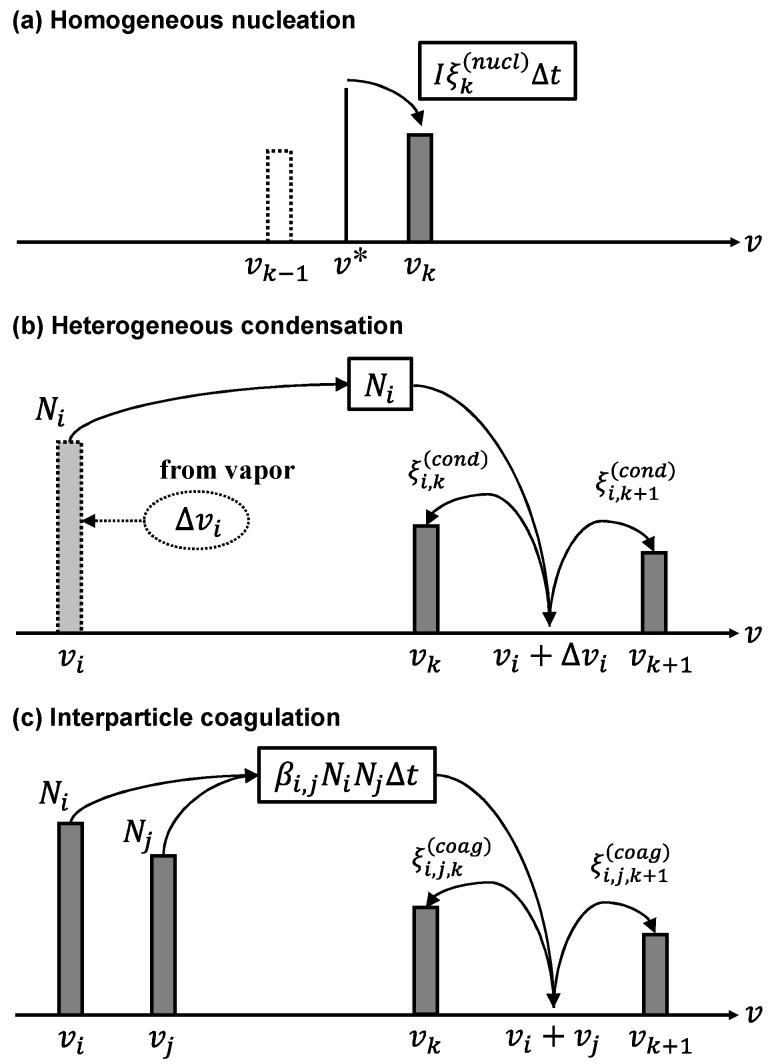
Illustrations depicting computations of (**a**) homogeneous nucleation, (**b**) heterogeneous condensation, and (**c**) interparticle coagulation.

Figure 2a portrays a schematic image showing calculations for homogeneous nucleation. When material vapor is transported to the plasma tail, its temperature decreases along with decreased saturation pressure of the material. Consequently, the material vapor becomes supersaturated, which engenders homogeneous nucleation. Therein, the production rate of particles having volume $v_{k}$ by homogeneous nucleation is written as

(3) $${\dot{N}}_{k}^{\left(nucl\right)}=I\xi_{k}^{\left(nucl\right)}\;\;\;\;\left(k=2,...,k_{max}\right)$$

here, I is the homogeneous nucleation rate [74] obtained as

(4) $$I=N_{s}^{2}S\sqrt{\frac{2\sigma }{\pi m_{1}}}\exp\left[\Theta -\frac{4\Theta^{3}}{27{\left(\ln S\right)}^{2}}\right]$$

where $N_{s}$ signifies the number density of the vapor atoms in the equilibrium state, σ stands for the surface tension, and $m_{1}$ denotes the mass of a monomer. Additionally, S expresses the supersaturation ratio defined as

(5) $$S=\frac{N_{1}}{N_{S}}=\frac{p_{1}}{p_{S}}$$

where $N_{1}$ and $p_{1}$ respectively represent the number density and pressure of vapor monomers, and $p_{S}$ denotes the saturation pressure. Furthermore, Θ stands for the dimensionless surface tension, which is given as

(6) $$\Theta =\frac{\sigma s_{1}}{k_{B}T}$$

where $s_{1}$ represents the monomer surface area, $k_{B}$ is Boltzmann’s constant, and T stands for the temperature. In Equation (3), $\xi_{k}^{\left(nucl\right)}$ represents the partition coefficient for discretized size nodes written as

(7) $$\xi_{k}^{\left(nucl\right)}=\left\{ \begin{array}{cc} \frac{v^{*}}{v_{k}} & \left(v_{k}\leq v^{*}<v_{k+1}\right)\\ \frac{v^{*}}{v_{1}} & \left(v^{*}<v_{1}\right)\\ 0 & \left(other\right) \end{array}\right.$$

here, $v^{*}$ represents the volume of a nucleus that is regarded as a particle having the critical diameter given as described in an earlier report [74],

(8) $$v^{*}=\frac{\pi }{6}{\left(\frac{4\sigma v_{1}}{k_{B}T\ln S}\right)}^{3}$$

Therefore, the critical diameter is equivalent to the diameter of a nucleus generated by homogeneous nucleation in this model.

### 3.3. Heterogeneous Condensation

Figure 2b represents a schematic image of calculation for heterogeneous condensation. When nuclei are generated by homogeneous nucleation, the material vapor is still supersaturated, which causes heterogeneous condensation. Therein, the net production rate of particles having volume $v_{k}$ by heterogeneous condensation can be written as

(9) $${\dot{N}}_{k}^{\left(cond\right)}=\overset{k_{max}}{\underset{i=2}{\sum }}\frac{(\xi_{i,k}^{\left(cond\right)}-\delta_{i,k})N_{i}}{\Delta t}\;\;\;\;\left(k=2,...,k_{max}\right)$$

here, $\xi_{i,k}^{\left(cond\right)}$ is the partition coefficient for heterogeneous condensation, which is given as

(10) $$\xi_{i,k}^{\left(cond\right)}=\{\begin{array}{cc} \frac{v_{k+1}-\left(v_{i}+\Delta v_{i}\right)}{v_{k+1}-v_{k}} & \left(v_{k}\leq v_{i}+\Delta v_{i}<v_{k+1}\right)\\ \frac{\left(v_{i}+\Delta v_{i}\right)-v_{k-1}}{v_{k}-v_{k-1}} & \left(v_{k-1}\leq v_{i}+\Delta v_{i}<v_{k}\right)\\ 0 & \left(other\right) \end{array}$$

In Equation (9), $\delta_{i,k}$ is Kronecker’s delta, and

(11) $$\delta_{i,k}=\{\begin{array}{cc} 1 & \left(i=k\right)\\ 0 & \left(i\neq k\right) \end{array}$$

The growth rate of particles having diameter $d_{i}$ is given as the following equation [64] modified from a general description as [75]

(12) $$\frac{\Delta v_{i}}{\Delta t}=2\pi d_{i}D_{1}v_{1}\left(N_{1}-N_{S_{i}}^{\prime }\right)\left[\frac{0.75\alpha \left(1+{\mathrm{Kn}}_{i}\right)}{\left(0.75\alpha \right)+0.2832\alpha {\mathrm{Kn}}_{i}+{\mathrm{Kn}}_{i}+{\mathrm{Kn}}_{i}^{2}}\right]$$

where $D_{1}$ is the diffusion coefficient of monomers [76], Kn is the Knudsen number defined as the ratio of the mean free path of gas to the particle radius, and α is the accommodation coefficient, which is set as 1.0 for this study. Additionally, $N_{S_{i}}^{\prime }$ denotes the number density of equilibrium vapor atoms corrected considering the surface curvature for node *i* as [77]

(13) $$N_{S_{i}}^{\prime }=N_{S}\exp\left(\frac{4\sigma v_{1}}{d_{i}k_{B}T}\right)$$

Particles with volume $v_{i}$ gain volume $\Delta v_{i}$. Consequently, they form new particles having volume $v_{i}+\Delta v_{i}$. The new particles are split into adjacent nodes under the mass conserving condition by Equation (10).

### 3.4. Interparticle Coagulation

Figure 2c portrays a schematic image of calculation for interparticle coagulation. The growing nanoparticles mutually collide and coagulate. When their temperature becomes higher than their melting point, the particles merge and form larger particles, thereby decreasing their own numbers. The net production rate of the particles at node *k* by interparticle coagulation can therefore be expressed as

(14) $${\dot{N}}_{k}^{\left(coag\right)}=\frac{1}{2}\;\overset{k_{max}}{\underset{i=2}{\sum }}\overset{k_{max}}{\underset{j=2}{\sum }}\xi_{i,j,k}^{\left(coag\right)}\beta_{i,j}N_{i}N_{j}-N_{k}\overset{k_{max}}{\underset{i=2}{\sum }}\beta_{i,k}N_{i}\;\;\;\;\left(k=2,...,k_{max}\right)$$

where $\beta_{i,j}$ represents the collision frequency function between two particles having volumes $v_{i}$ and $v_{j}$ covering a wide size range from the free molecular regime to the continuum regime [64]. The first term and the second term of the right-hand side in Equation (14) express the gain of node *k* by the collision between the particles at the other nodes *i* and *j*, and the loss of node *k* by the collision between the particles at node *k* and the particles at the other nodes, respectively. Particles with volume $v_{i}$ and $v_{j}$ collide and coagulate. They consequently form new particles with volume $v_{i}+v_{j}$. Figure 2c shows that the new particles are split into the adjacent nodes under the mass conserving condition presented in Figure 2. The partition coefficient for coagulation of size $\xi^{\left(coag\right)}$ is given as

(15) $$\xi_{i,j,k}^{\left(coag\right)}=\{\begin{array}{cc} \frac{v_{k+1}-\left(v_{i}+v_{j}\right)}{v_{k+1}-v_{k}} & \left(v_{k}\leq v_{i}+v_{j}<v_{k+1}\right)\\ \frac{\left(v_{i}+v_{j}\right)-v_{k-1}}{v_{k}-v_{k-1}} & \left(v_{k-1}\leq v_{i}+v_{j}<v_{k}\right)\\ 0 & \left(other\right) \end{array}$$

### 3.5. Vapor Consumption

The total number of material atoms is conserved through conversion from vapor to particles. The population balance equation of the material vapor is calculated simultaneously considering vapor atom consumption by nucleation and condensation:

(16) $$\frac{\Delta N_{1}}{\Delta t}=-\overset{k_{max}}{\underset{k=2}{\sum }}I\xi_{k}^{\left(nucl\right)}n^{*}-\overset{k_{max}}{\underset{i=2}{\sum }}\frac{N_{i}\Delta v_{i}}{v_{1}\Delta t}$$

In Equation (16), $n^{*}$ represents the number of monomers composing a thermodynamically neutrally stable nucleus. The number density of vapor atoms directly affects the rates of nucleation and condensation.

### 3.6. Melting Point Depression

Melting points of nanoscale particles are lower than those of bulk materials [78]. This melting point depression is considered in the present model. The melting point $T_{melt,\;k}$ of particles with diameter $d_{k}$ can be estimated as

(17) $$T_{melt,\;k}=T_{melt,\;bulk}\times \left(1-\frac{\varepsilon }{d_{k}}\right)\;\;\;\;\left(k=2,...,k_{max}\right)$$

**Figure 3:**
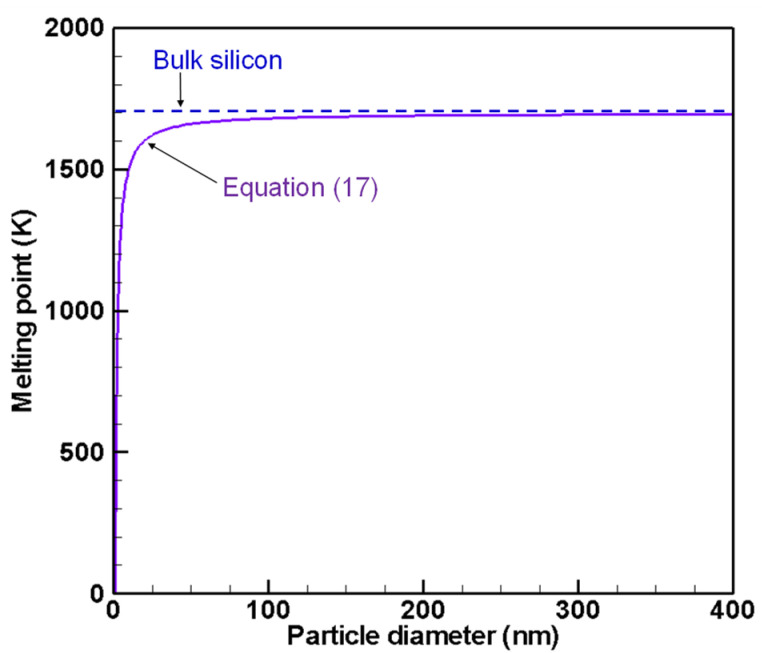
Melting points of bulk silicon and nano-size spherical silicon estimated by Equation (17).

Therein, $T_{melt,\;bulk}$ denotes the melting point of a bulk material. Parameter ε is a characteristic property determined by the solid and liquid surface energies and the bulk melting enthalpy. This study adopts the value of ε = 1.16 nm for silicon spherical particles [78]. Figure 3 depicts the melting points of bulk silicon and nanoscale spherical silicon estimated by Equation (17). The present model treats a particle with temperature lower than this depressed melting point as a solid body and does not calculate the interparticle coagulation if both particles are solid in computation.

### 3.7. Computational Conditions

Computations were performed under typical conditions at a small-type ICTP’s tail where the temperature decreases monotonically at 18 cooling rates ranging from 2.0 × 10^3^ K s^−1^ to 6.4 × 10^4^ K s^−1^. In response to these cooling rates, the time increment Δt for computations was set as 5.0 μs to 0.15625 μs, which gave sufficient resolution for the processes. Corresponding to the silicon feed rate of the experiment, it is assumed that, after complete evaporation by the plasma, the silicon vapor atoms are transported with the argon carrier gas. Therein, the molar fraction of silicon to argon is set to 1.257% at 2700 K as the starting condition of the computations. The material properties of silicon were referred from a textbook used for this field [79].

## 4. Results and Discussion

### 4.1. Experiment Results and Model Validation

**Figure 4:**
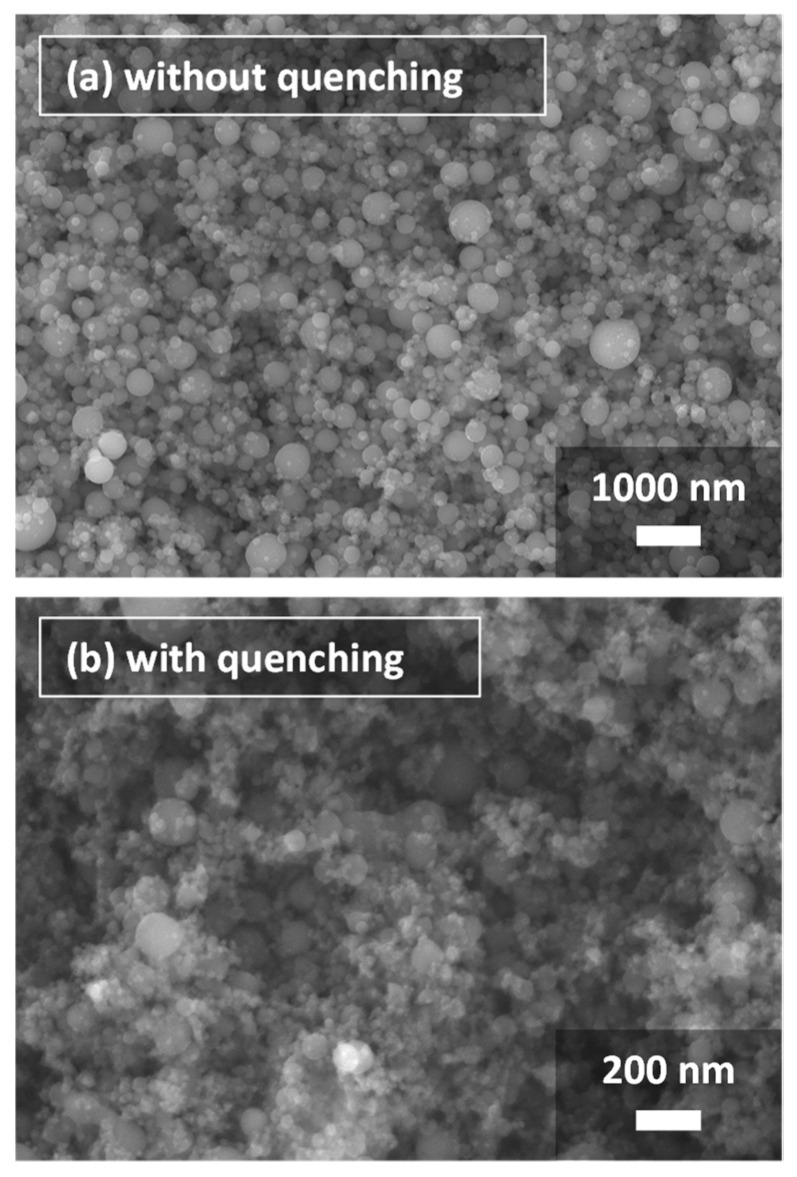
SEM images of silicon nanoparticles obtained through experiments (**a**) without and (**b**) with quenching.

**Figure 5:**
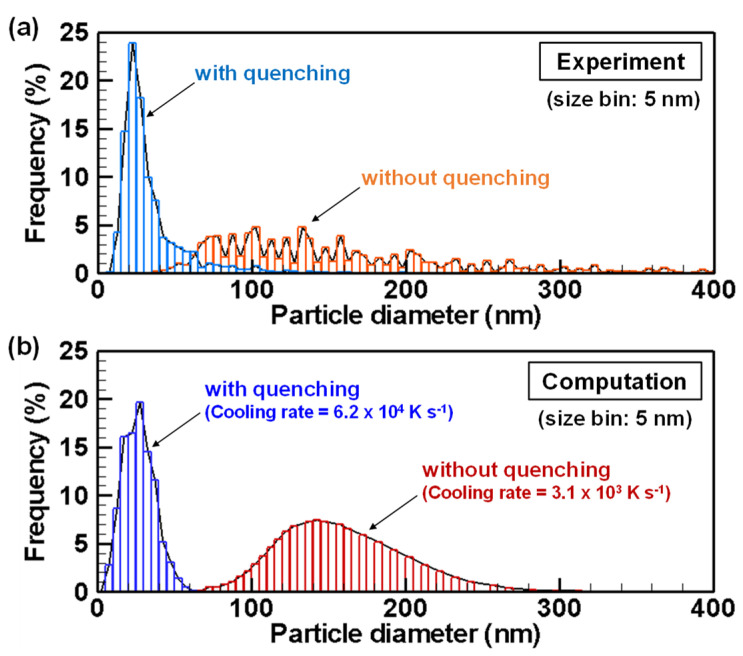
Size distributions of silicon nanoparticles obtained from (**a**) experiments and (**b**) computations.

Figure 4 depicts SEM images of silicon particles obtained with and without quenching. The primary particles are spherical particles having nanometer-range diameters. Apparently, the particles obtained with quenching are much smaller than those without quenching. Figure 5 shows the primary particle size distributions, which quantitatively indicate the same tendency in the experiments and the computations. With quenching, the size distributions exhibit smaller and narrower ranges. The experiment without quenching obtained mean diameter of 150 nm and standard deviation of 82 nm. The experiment with quenching obtained the mean diameter of 27 nm and standard deviation of 9 nm. On the other hand, computation with the cooling rate of 3.1 × 10^3^ K s^−1^ obtained mean diameter of 149.5 nm and standard deviation of 39.7 nm. Computation with the cooling rate of 6.2 × 10^4^ K s^−1^ obtained mean diameter of 26.8 nm and standard deviation of 9.98 nm. Those computation results agree to an acceptable degree with the experimentally obtained results. Therefore, in this report, the processes with the cooling rates of 6.2 × 10^4^ K s^−1^ and 3.1 × 10^3^ K s^−1^ are discussed as conditions with and without quenching, respectively.

In spite of that agreement, the results include some differences among them. The causes of the differences are discussed hereinafter. In general, experiments of practical material processing by thermal plasmas inherently include errors because of temporal fluctuations and spatial non-uniformity of temperature and flow fields, as reported from experimental studies [80,81] and earlier computational studies [26,30,82,83,84,85]. Such turbulent-like fluid-dynamic behaviors affect not only the evaporation of precursory particles [85,86] but also nanoparticle formation and transport [21,28,29]. Indeed, in the experiment reported herein, some large unvaporized precursor particles were found. On the other hand, the present numerical demonstrations were conducted under ideal conditions assuming complete evaporation of the precursor particles and nanoparticle growth at linear cooling rates. The electric charge might also affect the nanoparticle growth, even in the region downstream from the plasma tail [73]. However, that effect was neglected in the present computation. Recent studies conducted by computation [87,88] and experimentation [89,90] have demonstrated that ion transport by cataphoresis affected the material vapor distribution in and around plasma.

In addition, the surface tension of bulk silicon was used to calculate homogeneous nucleation by Equation (4) and the critical size by Equation (8). The diameters of major nuclei generated in this study were estimated as below a few nanometers, for which the surface tension of the bulk material might have not been valid. Validated data for such small sizes were unavailable. Therefore, bulk silicon data were used. It is noteworthy that numerical calculations of homogeneous nucleation rates and critical sizes inherently lead to errors because of this limitation. Nevertheless, Equations (4) and (8) are still accepted as the most reliable equations available.

It is noteworthy that nodal discretization of the size space introduces errors. To reduce error accumulation, the large number of nodes with small geometric spacing factor *f_q_* in Equation (1) should be used, as discussed in an earlier report of the relevant literature [66]. In the present study, several combinations of the node number and geometric spacing factor were tested. For instance, double the number of nodes led to changes of less than 1.5% in the particle size distribution. The set of an adequately large number of nodes and small geometric spacing factor was chosen to restrain error accumulation.

Heat generation by condensation was neglected. If all the vapor would be condensed, then the heat generation would be estimated as 1.1 W, which would heat the surrounding argon gas by approximately 24 K. Condensation occurred at 2500–2600 K. Therefore, the temperature increase effect was negligible. Even if no heat were transferred from silicon nanoparticles to argon gas, the temperature increase could be considerable and the temperature difference between them could be large. Actually, the condensation heat would be transferred to both and both temperatures would increase. If their temperature difference was large, then it would affect the growth rate. An additional model should be implemented to elucidate that point.

It might be significant to discuss about agglomerates as secondary larger particles as shown in SEM images of Figure 4. The rietveld analysis was performed from the X-ray diffraction (XRD) results. Calculated by the Halder-Wagner plot, the crystallite sizes were 73 nm with quenching and 74 nm without quenching. None of them were significantly smaller than the particle size obtained from the images. With quenching, the crystallite size is considered to be larger than the particle size due to the presence of unvaporized precursor particles. Meawhile, without quenching, the crystallite size is about half that of the particle size, but the effect of twinning during the solidification process from droplets cannot be ruled out. Therefore, in order to confirm these things experimentally, it is necessary to analyze the microstructure by Electron Back Scattered Diffraction (EBSD) measurements. A computational work treating aggregation was also reported using a different-type model [46], whereas the main scope of this study is more fundamental clarification of the vapor-to-particle conversion stage in quenching processes. The implementation of an aggregation model would be addressed in a further investigation.

The present model still has room for improvement. Nevertheless, the discussion presented above reinforces the assertion that the present computation validly predicts silicon nanoparticle growth processes in an ICTP system.

### 4.2. Implicit Mechanism of Collective Nanoparticle Growth

**Figure 6:**
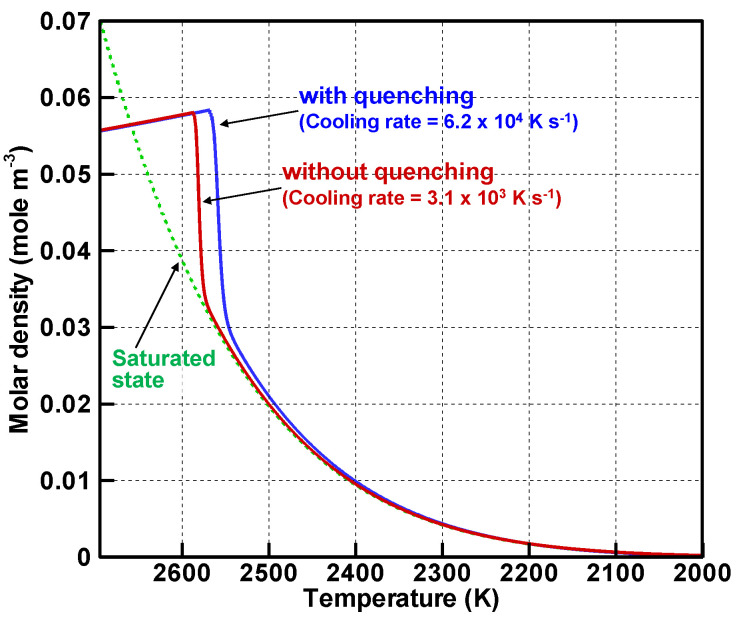
Evolutions of molar densities of vapor atoms and saturated states.

Figure 6 presents evolutions of molar densities of silicon vapor atoms and of the saturated state with and without quenching. The horizontal axes showing temperature are reversed corresponding to time progress with the temperature decrease. Both with and without quenching, the molar densities of the vapor atoms increase with the decrease in temperature. Then they exceed that of the saturated state. When the supersaturation ratios defined as Equation (5) reach the maximum values of 1.63 at 2586 K without quenching and 1.87 at 2567 K with quenching, the vapor atoms begin to decrease drastically. These decreases are caused by rapid conversion of the vapor atoms to nanoparticles.

**Figure 7:**
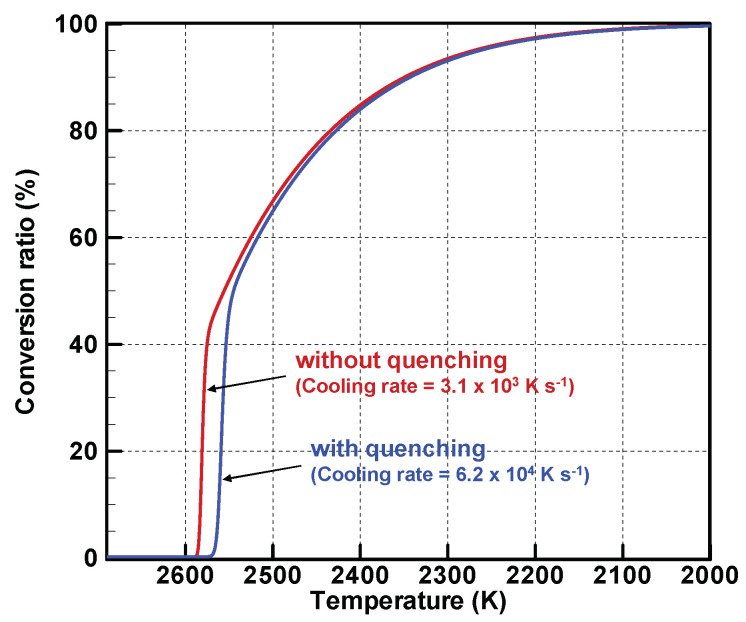
Evolutions of conversion ratios.

Figure 7 portrays evolutions of the conversion ratios, which represent how many vapor atoms have changed into nanoparticles. Approximately 40% and 50% of the vapor atoms are converted to nanoparticles at those rapid conversions. When the molar densities of the vapor atoms become slightly higher than that of the saturated state, as depicted in Figure 6, the conversions proceed moderately. For both conditions, 99% of the vapor atoms complete the conversions at 2100 K.

**Figure 8:**
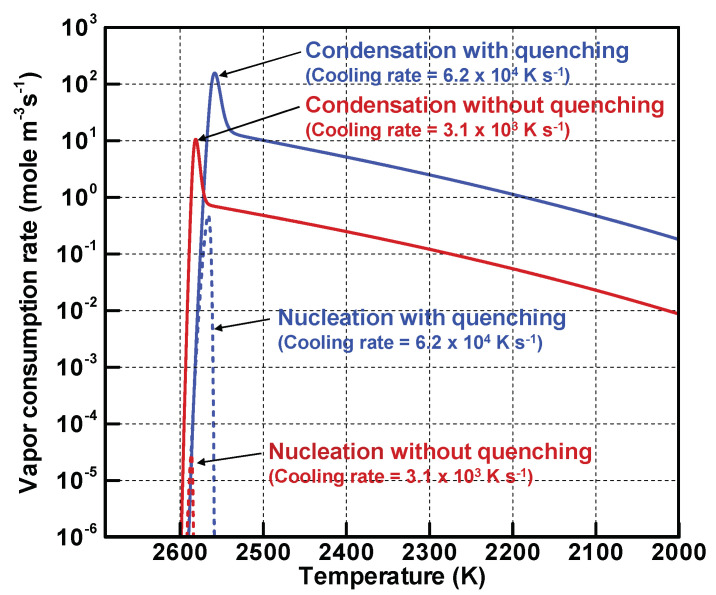
Evolutions of vapor consumption rates by homogeneous nucleation and heterogeneous condensation.

Figure 8 portrays the evolutions of vapor consumption rates by homogeneous nucleation and heterogeneous condensation. Without and with quenching, the rates of nucleation and condensation increase remarkably below 2600 K. When the number of nuclei increases and consequently the total surface area increases, vapor consumption by condensation becomes overwhelming. The consumption rate of condensation is approximately $4.0\times 10^{5}$ times as large as that of nucleation without quenching. On the other hand, that of condensation is approximately 340 times as large as that of nucleation with quenching. After the number densities of the vapor atoms decrease to that of the saturated state, the gradients of the consumption rates of condensation become more moderate, thereby leading to the moderately increasing curves in Figure 7.

**Figure 9:**
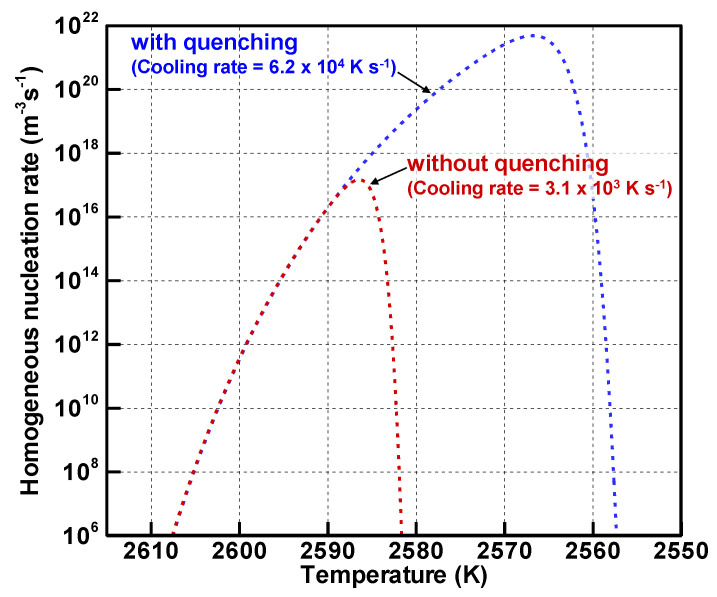
Evolutions of homogeneous nucleation rates.

Figure 9 depicts the evolutions of the homogeneous nucleation rates. As the vapor atoms become highly supersaturated, the nucleation rates increase and reach the highest values of $1.4\times 10^{17}$ m^−3^s^−1^ at 2586 K without quenching and $4.8\times 10^{21}$ m^−3^s^−1^ at 2567 K with quenching. After these moments, the nucleation rates decrease drastically.

**Figure 10:**
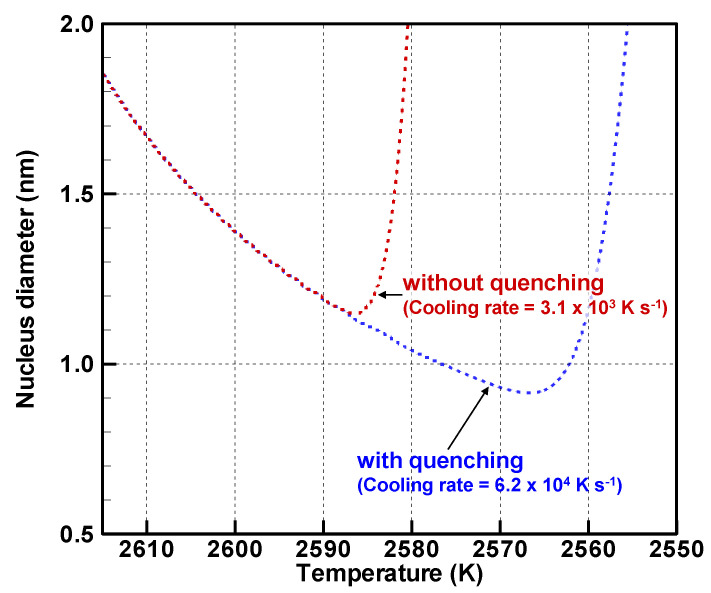
Evolutions of nucleus diameters.

Figure 10 displays the nucleus diameter evolution. The nucleus diameters take minimum values of 1.15 nm without quenching and 0.916 nm with quenching. The temperatures of those minimum values coincide with temperatures at which the homogeneous nucleation rates reach the highest values. Those results of Figure 9 and Figure 10 indicate that a larger number of smaller nuclei are generated under the quenching condition where the cooling rate is simply higher. The total numbers of the vapor atoms are the same with and without quenching. Then they are consumed mainly by heterogeneous condensation on the nuclei as presented in Figure 8. Therefore, because a larger number of the nuclei share the vapor atoms with quenching, fewer vapor atoms condense on a nucleus. As a result, the quenching condition will yield a larger number of smaller nanoparticles.

**Figure 11:**
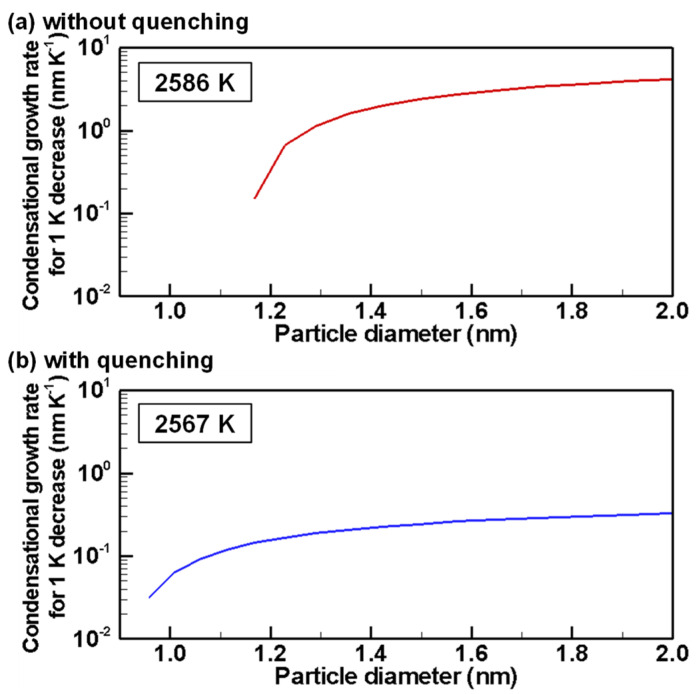
Instantaneous growth rates by heterogeneous condensation at temperatures of the highest homogeneous nucleation rate (**a**) without and (**b**) with quenching.

Figure 11 portrays the instantaneous growth rates by condensation for 1 K decrease for particle diameters around the nucleus diameters when the homogeneous nucleation rates reach the highest values. The growth rate without quenching is 0.16–4.15 nm K^−1^, which is much higher than that with quenching of 0.03–0.33 nm K^−1^. Moreover, the condition without quenching takes 323 μs, which is much longer than that with quenching of 16.1 μs for a 1 K decrease. Therefore, under the condition without quenching, the particles grow larger, whereas smaller nuclei are generated continuously over a longer period. Therefore, the condition without quenching produces a more dispersed size distribution with a larger standard deviation.

### 4.3. Evolution of Particle Size Distribution and Cooling Rate Dependency

**Figure 12:**
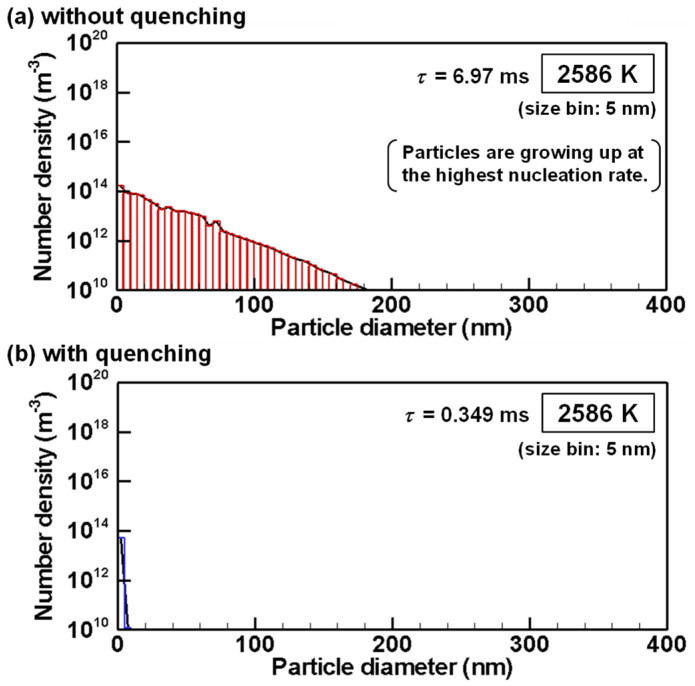
Size distributions of silicon nanoparticles at 2586 K (**a**) without and (**b**) with quenching.

**Figure 13:**
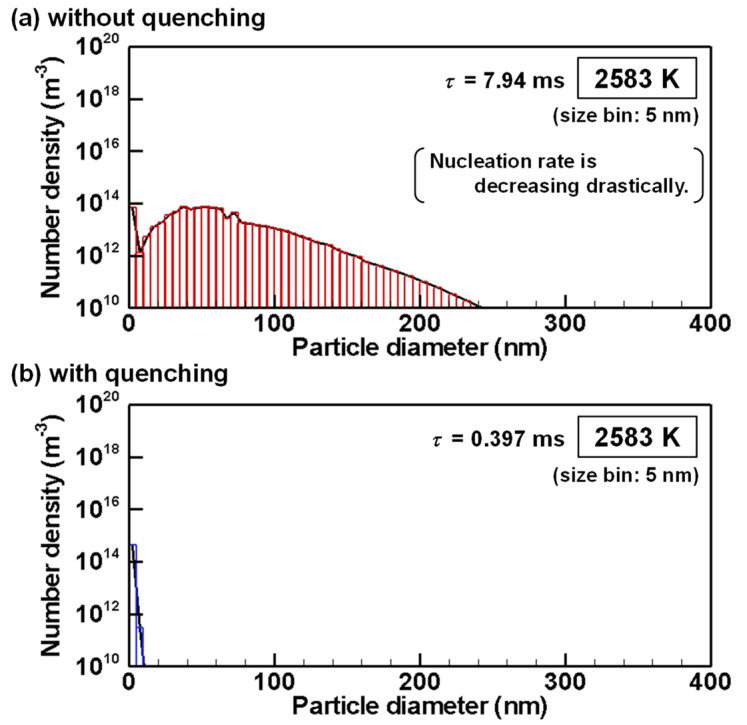
Size distributions of silicon nanoparticles at 2583 K (**a**) without and (**b**) with quenching.

**Figure 14:**
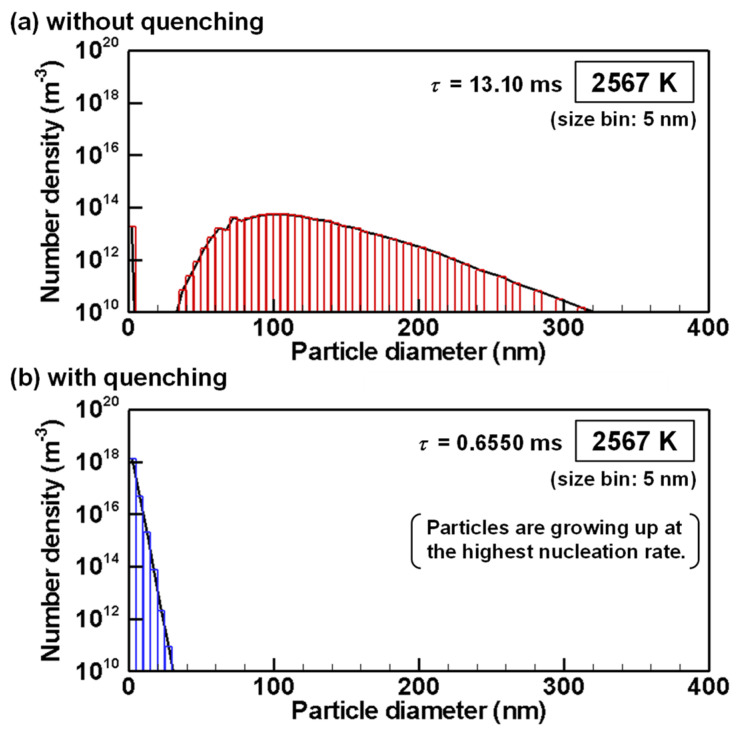
Size distributions of silicon nanoparticles at 2567 K (**a**) without and (**b**) with quenching.

**Figure 15:**
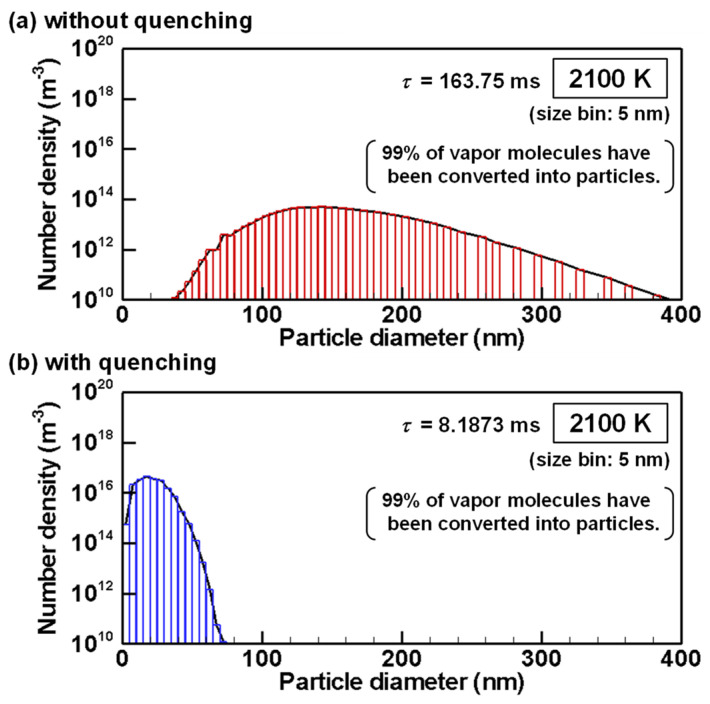
Size distributions of silicon nanoparticles at 2100 K (**a**) without and (**b**) with quenching.

**Figure 16:**
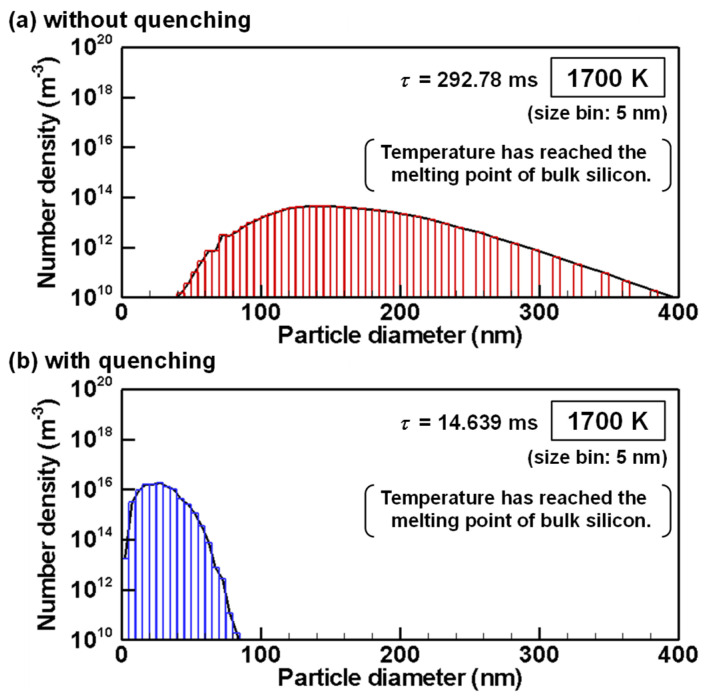
Size distributions of silicon nanoparticles at 1700 K (**a**) without and (**b**) with quenching.

The evolutions of particle size distributions with and without quenching are presented from Figure 12, Figure 13, Figure 14, Figure 15 and Figure 16 for representative temperatures. The corresponding movies are also presented in Video S1 as supplementary data. Therein, *τ* denotes the time elapsed after the homogeneous nucleation rate exceeded $1.0\times 10^{6}$ m^−3^s^−1^. It is noteworthy that one text [77] states that “particle formation can be conveniently observed experimentally” for this value. The conditions with and without quenching have different *τ* at the same temperature because the rates of temperature decrease differ.

Figure 12a shows that, at 2586 K, when the condition without quenching exhibits the highest nucleation rate, particles smaller than 5 nm present the largest number, whereas particles larger than 100 nm have already grown. On the other hand, the condition with quenching has only particles smaller than 5 nm as presented in Figure 12b.

Figure 13a shows that, at 2583 K, when the homogeneous nucleation rate is decreasing drastically, the particles have a bimodal distribution with two peaks under the condition without quenching because the particle growth changes from the nucleation dominant mode to the condensation dominant mode as indicated in Figure 8 and Figure 9. For Figure 13b, the particle growth is still in the nucleation dominant mode under the condition with quenching.

Figure 14b shows that, at 2567 K, when the condition with quenching offers the highest nucleation rate, the particles are still smaller than 30 nm. Particles smaller than 5 nm have the largest number. On the other hand, in Figure 14a, most particles have grown to larger particles under the condition without quenching. Particles smaller than 5 nm remain because the growth rate by heterogeneous condensation is lower for smaller particles because of their larger Knudsen numbers as indicated by Equation (12) and depicted in Figure 11. Those smaller nanoparticles will collide and merge with larger nanoparticles more frequently, thereby decreasing their numbers slowly.

Figure 15a,b show unimodal size distributions without and with quenching at *τ* = 163.75 ms and *τ* = 8.1873 ms, respectively. At these moments, the temperatures are 2100 K identically and 99% of the vapor atoms have already been converted into nanoparticles as shown in Figure 7. Therefore, after these moments, the nanoparticles grow by interparticle coagulation.

Figure 16a,b show size distributions obtained without and with quenching at 1700 K which is the melting point of bulk silicon. As depicted in Figure 3, the melting point of silicon nanoparticles are lower than bulk silicon. Therefore, the nanoparticles are still liquid and keep growing by interparticle coagulation. Without quenching, the product consists of particles large than 40 nm as shown in Figure 16a. Those nanoparticles will solidify and finish coagulation growth soon because the melting points for large nanoparticles are slightly lower than bulk silicon. On the other hand, with quenching, the product still includes many particles smaller than 40 nm as shown in Figure 16b. Due to the considerable depression of the melting points, their coagulation growth will progress longer. In addition, Figure 3 tells that the growing particles will start to solidify from large ones to small ones and eventually reach their final states. It is noteworthy that these size distributions in Figure 16 at 1700 K are not much different from those in Figure 15 at 2100 K, which means that growth by interparticle coagulation is much slower than that by heterogeneous condensation.

**Figure 17:**
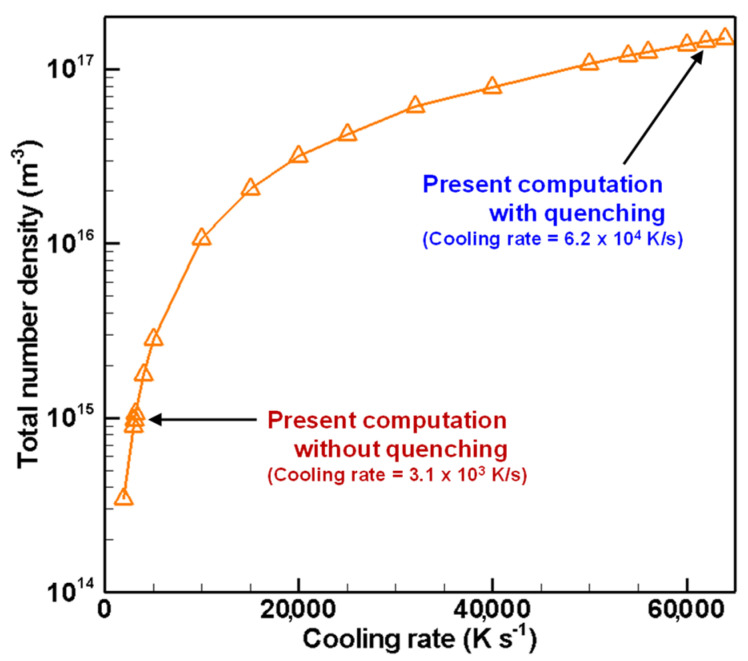
Cooling rate dependence on total number density of silicon nanoparticles.

**Figure 18:**
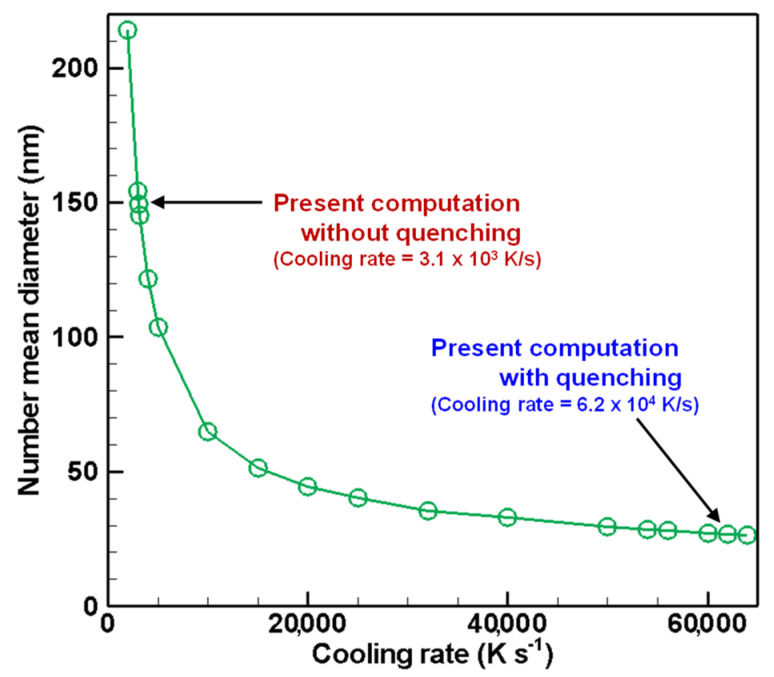
Cooling rate dependence on number mean diameter of silicon nanoparticles.

**Figure 19:**
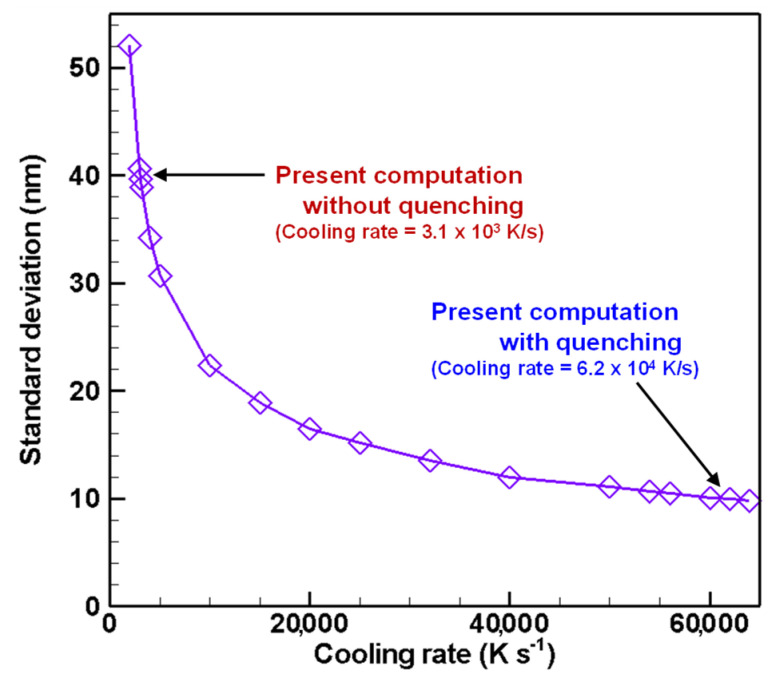
Cooling rate dependence on standard deviation of silicon nanoparticles.

Figure 17, Figure 18 and Figure 19 depict the cooling rate dependence on the total number density, the number mean diameter, and the standard deviation of the finally obtained silicon nanoparticles, respectively. A higher cooling rate can lead to higher total number density, smaller size, and smaller standard deviation. In an actual fabrication process, attention must be devoted to the fact that cooling rate control changes those obtained quantities simultaneously. It is therefore interesting that those quantities have no linear dependence on the cooling rate. These results suggest that quenching in thermal plasma fabrication is effectual, but it has limitations for controlling nanoparticle characteristics.

## 5. Conclusions

Quenching effects on the growth process and size distribution of silicon nanoparticles in the typical range of cooling rates at a thermal plasma tail were investigated computationally using a nodal-type model. Results suggest that the size distribution evolves temporally with simultaneous homogeneous nucleation, heterogeneous condensation, interparticle coagulation, and melting point depression. Furthermore, silicon nanoparticle fabrication was demonstrated experimentally with and without quenching. Those experiment results support the validity of the numerically obtained size distributions.

The implicit mechanism of the collective growth processes of silicon nanoparticles was revealed. When the vapor atoms became highly supersaturated, they decreased drastically by rapid conversion of the vapor atoms to nanoparticles. At these rapid conversions, approximately 40% and 50% of the vapor atoms were converted to nanoparticles. When the number of nuclei increased by nucleation and the total surface area consequently increased, vapor consumption by condensation became overwhelming. A larger number of smaller nuclei were generated under the quenching condition where the cooling rate was higher. Therein, fewer vapor atoms condensed on a nucleus because more nuclei shared the vapor atoms. As a result, the quenching condition yielded a larger number of smaller nanoparticles. After most of the vapor atoms were converted into particles, the particles grew by interparticle coagulation much more slowly than heterogeneous condensation.

As the cooling rate was increased, the total number density increased, the size decreased, and the standard deviation increased. Control of the cooling rate changed those quantities simultaneously. Furthermore, those quantities were found to have no linear dependence with the cooling rate. These results suggest that quenching in thermal plasma fabrication was effectual but that it had limitations for controlling nanoparticle characteristics. A sensitivity analysis for the individual effects on those quantities in respect to the cooling rate would be further exploration of the observed “no linear dependence”.

## Figures and Tables

**Table 1:** Computational growth models based on aerosol representation.

	Model Type A [51,54,55,56]	Model Type B [26,27,28,29,57,58]	Model Type C [39,40,42,43,44,45,46,47,59,60,61,62]	Model Type D [37,48,49,53,63,64,65,66,67,68,69,70,71,72,73]
Nucleation	considered	considered	considered	considered
Condensation	considered	considered	considered	considered
Coagulation	not considered	considered	considered	considered
Size distribution	any	mono-disperse	lognormal	any
Mathematical description	simpler	simpler	more complex	more complex
Computational costs	higher	lower	lower	higher

## Supplementary Materials

The following are available online at https://www.mdpi.com/article/10.3390/nano11061370/s1, Video S1: Evolutions of size distributions of silicon nanoparticles (a) without and (b) with quenching. *t*_0_ denotes the time at which the homogeneous nucleation rate exceeded $1.0\times 10^{6}$ m^−3^s^−1^: *t*_0_ = 29.8032 ms without quenching and *t*_0_ = 1.4902 ms with quenching. *τ* is defined as the time elapsed after *t*_0_.
